# A dataset for emotional reactions and family resilience during COVID-19 isolation period among Indonesian families

**DOI:** 10.1016/j.dib.2020.105946

**Published:** 2020-06-30

**Authors:** Maulana Rezi Ramadhana

**Affiliations:** School of Communication and Business, Telkom University

**Keywords:** Family resilience, Emotion, COVID-19, Parents, Indonesia

## Abstract

This study presents a survey dataset describing families’ conditions during the COVID-19 isolation period obtained from individuals who serve as parents. A survey was conducted to measure the family's positive or negative emotional reactions and the degree of their resilience. The data were categorized into age, sex, type of family, family size, length of marriage, family's environment, and family COVID-19 status. The samples were gathered from 365 parents of Indonesian students who were willing to fill an online questionnaire. SPSS v.23.0 was used to carry out descriptive statistics and intercorrelations. Additional results from chi-square analyses are available as supplemental tables in the Mendeley repository.

Specifications table

**Table:** 

Subject	Psychology
Specific subject area	Communication Psychology, Family Communication and Resilience
Type of data	Table
How data were acquired	Survey questionnaire (questionnaire included in Mendeley repository)
Data format	Raw Data, Analyzed Statistical Data
Parameters for data collection	Respondents were parents. The data were categorized into several groups, based on their demographic information such as gender, age, sex, the types of family, family size, the length of the marriage, family space, and, and family's COVID-19 status.
Description of data collection	The data were collected from April to May 2020. The questionnaire was distributed online to 450 families in Bandung, West Java. 365 families filled out the questionnaires and were considered as valid responses
Data source location	West Java, Indonesia 7.0909° S, 107.6689° E
Data accessibility	Data which contained in this article are accesible in Mendeley Data: https://doi.org/10.17632/srzztj733v.4

## Value of the data

• The data are useful to depict families’ resilience (e.g. meaning-making, positive outlook, transcendence, flexibility, connectedness, social resources, communication, emotional disclosure, and problem-solving) and emotional problems that emerge during COVID-19 isolation period in Indonesia.
• The data benefits social workers and local government in designing family procedures for fighting COVID-19 pandemic (for instance, developing a family communication strategy, enhancing family health, reducing family stress, strengthening family resilience during COVID-19), and controlling well-being index during the COVID-19 recovery period to strengthen family resilience.
• This study can be replicated in other countries with different family cultures and analyzed further to find out other variables of family resilience based on other family demography.

## 1 Data description

The dataset provides insight into the family's resilience [1,2], and the family's emotional reaction during the COVID-19 isolation period [3]. This data mainly reports the questionnaire (questionnaire included in Mendeley repository) and raw data (for each subject, response toward items, and variable score; see Mendeley data). In addition, it also reveals demographic statistics of the sample (e.g. age, sex, types of family, family size, length of the marriage, family's environment, and family's COVID-19 status, see Table 1). The data describes positive emotion, negative emotion, and family resilience (Table 2), the relationship between positive and negative emotion (Table 3), family resilience and positive emotion (Table 4), and negative emotion (Table 5). The demographic data of the variable and SPSS syntax to calculate the mean of the variables are available (see Mendeley Data).

**Table 1:** Family demographics: age, sex, family type, family size, length of marriage, family's environment, family COVID-19 status (N=365).

Variables	Category	Frequency	
		Number	Percent
Age	less than 40 years old	14	3.8
	41–45 years old	75	20.5
	46–50 years old	89	24.4
	51–55 years old	83	22.7
	more than 56 years old	104	28.5
Sex	Male	174	47.7
	Female	191	52.3
Family Type	Nuclear family	329	90.1
	Joint family	12	3.3
	Extended family	24	6.6
Family Size	One people	0	0
	Two people	16	4.4
	Three people	30	8.2
	Four people	132	36.2
	Five people	127	34.8
	Six people	33	9.0
	Seven people	16	4.4
	Eight people	5	1.4
	More than eight people	6	1.6
Length of marriage	Less than 5 years	36	9.9
	5–10 years	64	17.5
	10–15 years	64	17.5
	15–20 years	32	8.8
	20–25 years	79	21.6
	More than 25 years	90	24.7
Family living area	Rural	55	15.1
	Suburban	112	30.7
	Urban	198	54.2
Family COVID-19 status	Asymptomatic person	305	83.6
	Persons under monitoring	27	7.4
	patients under supervision	32	8.8

*Note:* The seven family demographic variables were coded in data as Age (1-less than 40 years old, 2–41–45 years old, 3–46–50 years old, 4–51–55 years old, 5-more than 56 years old), Sex (1-male, 2-female), Family type (1-Nuclear, 2-Joint, 3-Extended), Family size (1-one people, 2-two people, 3-three people, 4-four people, 5-five people, 6-six people, 7-seven people, 8-eight people, 9-more than eight people), Length of marriage (1-less than 5 years, 2-5-10 years, 3-10-15 years, 4-15-20 years, 5-20-25 years, 6-more than 25 years), Family living area (1-rural, 2-suburban, 3-urban), Family COVID-19 status (1-asymptomatic person, 2-persons under monitoring, 3-patients under supervision).

**Table 2:** Descriptive statistic by positive emotion, negative emotion, and family resilience variables.

Variables	Mean	SD	Range
Positive emotion			
Love	2.80	.86	1.00–3.48
Gratitude	2.93	.91	1.00–3.92
Happiness	3.34	.88	1.00–4.08
Entertainment	3.07	.91	1.00–3.96
Satisfaction	2.84	.90	1.00–3.76
Relief	2.76	.92	1.00–3.77
Negative emotion			
anxiety	2.50	.95	1.00–4.02
Sadness	2.25	.95	1.00–3.91
Anger	2.26	.95	1.00–3.95
Fear	2.22	.94	1.00–4.09
Boredom	2.14	.94	1.00–3.93
Despair	2.14	.94	1.00–4.00
Family resilience			
Making meaning	3.76	.74	1.00–4.58
Positive outlook	3.84	.73	1.00–4.70
Transcendence	3.57	.73	1.53–4.41
Flexibility	3.66	.77	1.00–4.56
Connectedness	3.49	.83	1.00–4.38
Social resources	3.73	.70	1.69–4.69
Communication	3.68	.82	1.00–4.71
Open emotional	3.32	.80	1.00–4.40
Problem solving	3.59	.84	1.00–4.53

**Table 3:** Correlation among positive and negative emotion during COVID-19.

Negative emotion	Positive emotion					
	Love	Gratitude	Happiness	Entertainment	Satisfaction	Relief
Anxiety	-.066	-.007	-.167**	-.095	-.028	-.024
Sadness	-.079	.069	.003	-.046	.071	-.005
Anger	-.091	-.043	-.094	-.066	.061	-.052
Fear	-.112*	-.077	-.157	-.064	-.004	-.043
Boredom	.017	-.019	.088	.012	.023	-.049
Despair	-.013	-.032	.041	-.013	.042	-.013

*Notes:* * p < .05 ** < p .01

**Table 4:** Correlation among family resilience aspect and positive emotion during COVID-19.

Family Resilience	Positive emotion					
	Love	Gratitude	Happiness	Entertainment	Satisfaction	Relief
Making meaning	.227**	.132*	.126*	.195**	.139**	.188**
Positive outlook	.156**	.156**	.089	.175**	.144**	.193**
Transcendence	.269**	.163**	.188**	.306**	.220**	.303**
Flexibility	.175**	.150**	.116*	.259**	.159**	.226**
Connectedness	.246**	.092	.145**	.273**	.117*	.218*
Social resources	.144**	.159**	.080	.206**	.095	.156**
Communication	.189**	.076	.152**	.266**	.064	.233**
Open emotional	.291**	.066	.210**	.324**	.179**	.291**
Problem solving	.341**	.122*	.213**	.318**	.163**	.297**

*Notes:* * p < .05 ** < p .01

**Table 5:** Correlation among family resilience aspect and negative emotion during COVID-19.

Family Resilience	Negative emotion					
	anxiety	sadness	anger	fear	boredom	despair
making meaning	-.089	-.063	-.114*	-.074	-.051	-.053
positive outlook	-.109*	-.036	-.096	-.058	-.025	-.016
transcendence	-.137**	-.096	-.157**	-.118*	-.055	-.046
flexibility	-.152**	-.087	-.126*	-.078	-.045	-.038
connectedness	-.125*	-.146**	-.145**	-.089	-.007	-.089
social resources	-.112*	.000	-.103*	-.029	-.024	-.086
communication	-.128*	.103*	-.122*	-.028	-.060	-.053
open emotional	-.122*	.077	-.199**	-.115*	-.053	-.072
problem solving	-.134*	.045	-.152**	-.079	-.021	-.055

*Notes:* * p < .05 ** < p .01

## 2 Experimental design, materials and methods

Participants were parents (father or mother) of students who were performing self-isolation at home. They were selected using a simple random sampling technique. Demographic data such as age, sex, type of family, family size, length of the marriage, family's environment, and family's COVID-19 status were employed. Out of 450 parents, 372 parents gave their responses, and 365 of them filled the questionnaire. During the survey, participants were well informed about the confidentiality of their responses. The data were collected in 21 days (April-May 2020) during the COVID-19 isolation period using questionnaires. The questionnaires were adapted from family resilience literature [4] (see file ‘questionnaire’ and ‘variable’ in Mendeley repository). To perform the survey, respondents were asked to answer all items. Also, informed consent was obtained from all participants. The data concerned with family demography, positive and negative emotional reaction, and family resilience during the COVID-19 isolation period. The questionnaires were designed by following a Likert scale system, requiring the participants to rate items from 1 (strongly disagree) to 5 (strongly agree). After that, SPSS v.23.0 was used to carry out descriptive statistics, a median test, Kruskal-Wallis, and a correlational test.

Family demography consisted of types of the family [5]. Family's COVID-19 status was categorized based on the Indonesian government's policy, namely ‘asymptomatic person’, ‘persons under monitoring’, and ‘patients under supervision’ [6]. Another category that was added was deceased family members. The family's environment was measured using the category of the residential area [7]. Family size and length of the marriage were shown by using numbers. Meanwhile, emotion represents condition [8], predicts reaction [9], and thought and action that emerges [10,11]. The emotion was distinguished using the ‘positive’ and ‘negative’ category [12]. The former involved love [13], satisfaction [14], happiness [15], gratitude [16], entertainment [17], and relief [18]. Meanwhile, the latter included anger and fear [17], boredom [19], anxiety [20], sadness [21] and despair [22]. Positive and negative emotional reactions were measured using questionnaire items starting with the phrase “During COVID-19 isolation period,...”.

Family resilience in this data covered three aspects, namely the family belief system, patterns of family organization, and communication [4]. The belief system was assessed using three aspects, covering meaning-making (4 items; e.g., "We view distress with our situation as common, understandable", positive view (4 items, e.g.: "We encourage each other and build" on our strengths"), and transcendence (5 items, e.g., "We share important values and life goals that help us rise above difficulties "). Further, patterns of the family organization were assessed from three aspects such as flexibility (3 items; e.g. "We are flexible in adapting to new challenges"), connectedness (2 items, e.g., "We can count on family members to help each other in difficulty"), and social resources (4 items, e.g., "We can access community resources to help our families through difficult times"). Finally, communication was assessed in three aspects such as clarity (2 items; e.g., "We try to clarify information about our stressful situation and our options"), emotional disclosure (4 items, e.g., "We can express our opinions and be truthful with each other"), and collaborative problem-solving (4 items, e.g., " We plan and prepare for the future and try to prevent crises").

## Ethics Statement

This data serves as information about students’ conditions during the COVID-19 isolation period. Written informed consent was obtained from the participants.

## Declaration of Competing Interest

The authors declare that they have no known competing financial interests or personal relationships which have, or could be perceived to have, influenced the work reported in this article.

## Appendix Supplementary materials

Image, application 1
